# Supplementary material for: Estimation of Tinnitus-Related Socioeconomic Costs in Germany
Source: Int J Environ Res Public Health. 2022 Aug 22;19(16):10455. doi: 10.3390/ijerph191610455 (PMC9407899; doi:10.3390/ijerph191610455)
Supplement: Supplementary file 1 [file ijerph-19-10455-s001.zip › ijerph-1831753-supplementary.pdf]

Supplementary Table S1. Mean health care costs separated for each scenario

Visit at specific medical personal or service	Cost scenario	public health care insurance costs (€)	Private health care insurance costs (€)
General practitioner (GP)	1	27.20	
	2	17.68	
	3	9.52	
	4	0	
	5		33.52
	6		40.71
House call GP	1	58.71	
	2	49.19	
	3	41.03	
	4	31.51	
	5		51.85
	6		68.43
Emergency service	1	21.10	
	2		44.01
	3		46.34
	4		56.83
ENT specialist	1	67.38	
	2	45.57	
	3		74.62
ENT clinic	1	97.50	
	2		138.05
Neurologist	1	87.45	
	2	75.06	
	3		212.06
Audiologist	1	255.00	
	2		500.00
Psychiatrist	1	36.15	
	2	14.72	
	3		67.04
Psychotherapist	1	86.55	
	2	86.20	
	3		107.69

Occupational therapist	1	65.72	
	2		118.30
Dentist	1	19.49	
	2	125.43	
	3		23.66
	4		180.86
Physiotherapist	1	30.64	
	2		51.55
Social worker	1	8.59	8.59
Medical officer	1	6.75	6.75

Supplement: Questionnaires

Tinnituszentrum

Fragebogen zu vorangegangenen medizinischen Untersuchung

I

Liebe Forschungsteilnehmer,

Sie nehmen an der Studie über Tinnitus-Symptome und möglichen Behandlungen teil. Im Rahmen dieser Forschung möchten wir überprüfen, welche medizinische Versorgung Sie in den letzten Monaten in Bezug auf Ihre Tinnitus-Symptome erhalten haben. Daher wird auch gefragt, welche Leistungen des Gesundheitswesens Sie in Anspruch genommen oder welchen Behandlungen Sie sich in den vergangenen zwei Monaten unterzogen haben.

Versuchen Sie, so viele Fragen wir möglich zu beantworten.
Es gibt keine richtigen oder falschen Antworten.

Questionnaire on previous medical examination

Dear research participants,
You are taking part in the study on tinnitus symptoms and possible treatments. As part of this research, we would like to check what healthcare you have received in the last few months regarding your tinnitus symptoms. Therefore, we also ask what health care services you have used or what treatments you have undergone in the past two months.

Try to answer as many questions as possible.
There are no right or wrong answers.

1. Wie oft haben Sie in den letzten drei Monaten Ihren Arzt aufgrund der Tinnitussymptome aufgesucht?

Anzahl der Besuche: ...

wenn Sie keinen Kontakt gehabt haben, geben Sie bitte 0 ein.

how often have you visited your doctor in the last three months because of the tinnitus symptoms?
Number of visits: ...
if you have had no contact, please enter 0.

nur, wenn Sie den Arzt aufgesucht haben:

2. Wie sind Sie in der Regel zur Praxis gekommen?

- o Auto
- o Fahrrad oder zu Fuß
- o Öffentliche Verkehrsmittel
- o andere

only if you have visited the doctor:
how did you usually get to the practice?
o Car
o Bicycle or on foot
o Public transport
o Other

3. Wie oft haben Sie in den letzten drei Monaten aufgrund der Tinnitussymptome einen Hausbesuch Ihres Arztes erhalten?

Anzahl der Besuche: ...

wenn Sie keinen Kontakt gehabt haben, geben Sie bitte 0 ein.

in the last three months, how often did you receive a home visit from your doctor because of the tinnitus symptoms?
Number of visits: ...
if you have had no contact, please enter 0.

4. Wie oft haben Sie in den letzten drei Monaten den Arzt oder eine Praxis aufgrund der Tinnitusbeschwerden aufgesucht?

Anzahl der Besuche: ...

wenn Sie keinen Kontakt gehabt haben, geben Sie bitte 0 ein.

how often have you visited the doctor or a practice in the last three months because of the tinnitus complaints?
Number of visits: ...
if you have had no contact, please enter 0.

nur, wenn Sie Kontakt gehabt haben:

5. **Wie sind Sie in der Regel zum Arzt gekommen?**
    - o Auto
    - o Fahrrad oder zu Fuß
    - o Öffentliche Verkehrsmittel
    - o andere

only if you have had contact:
How did you usually get to the doctor?
o Car
o Bicycle or on foot
o Public transport
o Other

6. **Wie oft haben Sie in den letzten drei Monaten aufgrund der Tinnitusbeschwerden den ärztlichen Abend- und Wochenenddienst (Notdienst) aufgesucht?**

Anzahl der Besuche: ...

wenn Sie keinen Kontakt gehabt haben, geben Sie bitte 0 ein.

how often have you visited the medical evening and weekend service (emergency service) in the last three months because of the tinnitus complaints?
Number of visits: ...
if you have had no contact, please enter 0.

nur, wenn Sie Kontakt gehabt haben:

7. **Wie sind abends und am Wochenende meistens zum ärztlichen Notdienst gekommen?**
    - o Auto
    - o Fahrrad oder zu Fuß
    - o Öffentliche Verkehrsmittel
    - o andere

only if you have had contact:
how did you usually get to the emergency medical service in the evenings and at weekends?
o Car
o Bicycle or on foot
o Public transport
o Other

8. **Wie oft haben Sie in den letzten drei Monaten den Kontakt mit einem Physiotherapeuten oder Chiropraktiker aufgrund von Tinnitusbeschwerden gesucht?**

Anzahl der Besuche: ...

wenn Sie keinen Kontakt gehabt haben, geben Sie bitte 0 ein.

how often have you sought contact with a physiotherapist or chiropractor for tinnitus complaints in the last three months?
Number of visits: ...
if you have had no contact, please enter 0.

nur, wenn Sie Kontakt gehabt haben:

9. Wie sind Sie in der Regel zum Physiotherapeuten oder Chiropraktiker gekommen?

- o Auto
- o Fahrrad oder zu Fuß
- o Öffentliche Verkehrsmittel
- o andere

only if you have had contact:
How did you usually get to the physiotherapist or chiropractor?
o Car
o Bicycle or on foot
o Public transport
o Other

10. Wie oft hatten Sie in den letzten drei Monaten aufgrund von Tinnitusbeschwerden Kontakt mit einem Psychologen?

Anzahl der Besuche: ...

wenn Sie keinen Kontakt gehabt haben, geben Sie bitte 0 ein.

how often did you have contact with a psychologist in the last three months due to tinnitus complaints?
Number of visits: ...
if you have had no contact, please enter 0.

nur, wenn Sie Kontakt gehabt haben:

11. Wie sind Sie in der Regel zum Psychologen gekommen?

- o Auto
- o Fahrrad oder zu Fuß
- o Öffentliche Verkehrsmittel
- o andere

only if you have had contact:
How did you usually get to the psychologist?
o car
o Bicycle or on foot
o Public transport
o Other

12. Wie oft hatten Sie in den letzten drei Monaten aufgrund von Tinnitusbeschwerden Kontakt mit einem Sozialarbeiter?

Anzahl der Besuche: ...

wenn Sie keinen Kontakt gehabt haben, geben Sie bitte 0 ein.

how often have you had contact with a social worker in the last three months due to tinnitus complaints?
Number of visits: ...
if you have had no contact, please enter 0.

nur, wenn Sie Kontakt gehabt haben:

13. Wie sind Sie in der Regel zum Sozialarbeiter gekommen?

- o Auto
- o Fahrrad oder zu Fuß
- o Öffentliche Verkehrsmittel
- o andere

only if you have had contact:
how did you usually get to the social worker?
o car
o Bicycle or on foot
o Public transport
o Other

14. Wie oft hatten Sie in den letzten drei Monaten aufgrund von Tinnitusbeschwerden Kontakt mit einem Ergotherapeuten?

Anzahl der Besuche: ...

wenn Sie keinen Kontakt gehabt haben, geben Sie bitte 0 ein.

how often did you have contact with an occupational therapist in the last three months due to tinnitus complaints?
Number of visits: ...
if you have had no contact, please enter 0.

nur, wenn Sie Kontakt gehabt haben:

15. Wie sind Sie in der Regel zum Ergotherapeuten gekommen?

- o Auto
- o Fahrrad oder zu Fuß
- o Öffentliche Verkehrsmittel
- o andere

only if you have had contact:
how did you usually get to the occupational therapist?
o car
o Bicycle or on foot
o Public transport
o Other

16. Wie oft haben Sie in den letzten drei Monaten aufgrund der Tinnitusbeschwerden einen HNO-Arzt aufgesucht?

Anzahl der Besuche: ...

wenn Sie keinen Kontakt gehabt haben, geben Sie bitte 0 ein.

how often have you visited an ENT doctor in the last three months because of the tinnitus complaints?
Number of visits: ...
if you have had no contact, please enter 0

nur, wenn Sie Kontakt gehabt haben:

17. Wie sind Sie in der Regel zum HNO-Arzt gekommen?

- Auto
- Fahrrad oder zu Fuß
- Öffentliche Verkehrsmittel
- andere

only if you have had contact:
how did you usually get to the ENT doctor?
o Car
o Bicycle or on foot
o Public transport
o Other

18. Wie oft haben Sie in den letzten drei Monaten aufgrund der Tinnitusbeschwerden einen Zahnarzt aufgesucht?

Anzahl der Besuche: ...

wenn Sie keinen Kontakt gehabt haben, geben Sie bitte 0 ein.

how often have you visited a dentist in the last three months because of the tinnitus symptoms?
Number of visits: ...
if you have had no contact, please enter 0.

nur, wenn Sie Kontakt gehabt haben:

19. Wie sind Sie in der Regel zum Zahnarzt gekommen?

- Auto
- Fahrrad oder zu Fuß
- Öffentliche Verkehrsmittel
- andere

only if you have had contact:
how did you usually get to the dentist?
o car
o Bicycle or on foot
o Public transport
o Other

20. Wie oft haben Sie in den letzten drei Monaten aufgrund der Tinnitusbeschwerden einen Neurologen aufgesucht?

Anzahl der Besuche: ...

wenn Sie keinen Kontakt gehabt haben, geben Sie bitte 0 ein.

how often have you seen a neurologist in the last three months because of the tinnitus symptoms?
Number of visits: ...
if you have had no contact, please enter 0.

nur, wenn Sie Kontakt gehabt haben:

21. Wie sind Sie in der Regel zum Neurologen gekommen?

- o Auto
- o Fahrrad oder zu Fuß
- o Öffentliche Verkehrsmittel
- o andere

only if you have had contact:
how did you usually get to the neurologist?
o Car
o Bicycle or on foot
o Public transport
o Other

22. Wie oft haben Sie in den letzten drei Monaten aufgrund der Tinnitusbeschwerden einen Psychiater aufgesucht?

Anzahl der Besuche: ...

wenn Sie keinen Kontakt gehabt haben, geben Sie bitte 0 ein.

how often have you seen a psychiatrist in the last three months because of the tinnitus complaints?
Number of visits: ...
if you have had no contact, please enter 0.

nur, wenn Sie Kontakt gehabt haben:

23. Wie sind Sie in der Regel zum Psychiater gekommen?

- o Auto
- o Fahrrad oder zu Fuß
- o Öffentliche Verkehrsmittel
- o andere

only if you have had contact:
how did you usually get to the psychiatrist?
o Car
o Bicycle or on foot
o Public transport
o Other

24. Wie oft haben Sie in den letzten drei Monaten aufgrund der Tinnitusbeschwerden einen Betriebsarzt aufgesucht?

Anzahl der Besuche: ...

wenn Sie keinen Kontakt gehabt haben, geben Sie bitte 0 ein.

how often have you visited a company doctor in the last three months because of the tinnitus complaints?
Number of visits: ...
if you have had no contact, please enter 0.

nur, wenn Sie Kontakt gehabt haben:

24a. Wie sind Sie in der Regel zum Betriebsarzt gekommen?

- o Auto
- o Fahrrad oder zu Fuß
- o Öffentliche Verkehrsmittel
- o andere

only if you have had contact:
How did you usually get to the company doctor?
o Car
o Bicycle or on foot
o Public transport
o Other

25. Habend Sie in den letzten drei Monaten aufgrund der Tinnitusbeschwerden einen Spezialisten / eine Fachklinik aufgesucht?

- o Ja Name ______________________
  Fachrichtung ______________________
  Krankenhaus ______________________
- o Nein

have you visited a specialist / a specialist clinic in the last three months because of the tinnitus complaints?
O Yes Name ______________________
Subject ______________________
Hospital ______________________

o No

nur, wenn Sie Kontakt gehabt haben:

26. Wir oft haben Sie in den letzten drei Monaten aufgrund der Tinnitusbeschwerden einen Spezialisten / eine Fachklinik aufgesucht?

Anzahl der Besuche: ...

wenn Sie keinen Kontakt gehabt haben, geben Sie bitte 0 ein.

only if you have had contact:
in the last three months, how often have you visited a specialist / a specialist clinic because of the tinnitus complaints?
Number of visits: ...
if you have not had any contact, please enter 0.

nur, wenn Sie Kontakt gehabt haben:

27. **Wie sind Sie in der Regel zum Spezialisten / zur Fachklinik gekommen?**
    - o Auto
    - o Fahrrad oder zu Fuß
    - o Öffentliche Verkehrsmittel
    - o andere

only if you have had contact:
how did you usually get to the specialist / specialist clinic?
o Car
o Bicycle or on foot
o Public transport
o Other

28. **Wie oft haben Sie in den letzten drei Monaten aufgrund der Tinnitusbeschwerden einen Akustiker / Audiologen (im Krankenhaus oder in einem audiologischen Zentrum) aufgesucht?**

    Anzahl der Besuche: ...

    wenn Sie keinen Kontakt gehabt haben, geben Sie bitte 0 ein.

how often have you visited an acoustician / audiologist (in hospital or in an audiology centre) in the last three months because of the tinnitus complaints?
Number of visits: ...
if you have had no contact, please enter 0.

nur, wenn Sie Kontakt gehabt haben:

29. **Wie sind Sie in der Regel zum Akustiker / Audiologen gekommen?**
    - o Auto
    - o Fahrrad oder zu Fuß
    - o Öffentliche Verkehrsmittel
    - o andere

only if you have had contact:
how did you usually get to the acoustician / audiologist?
o Car
o Bicycle or on foot
o Public transport
o Other

30. Haben Sie aufgrund der Tinnitusbeschwerden in den letzten drei Monaten verschreibungspflichtige Medikamente genommen?

- Ja
- Nein

have you taken any prescription medication due to the tinnitus symptoms in the last three months?
o Yes
o No

nur, wenn Sie verschriebene Medikamente einnehmen

31. Welche Medikamente nehmen Sie? Bitte ankreuzen:

- Nitrazepam (Mogadon)
- Temazepam (Normison)
- Oxazepam (Seresta)
- Lormetazepam (Loramet)
- Zolpidem (Stilnoct)
- Alprazolam (Xanax)
- Lorazepam (Temesta)
- Diazepam (Valium)
- Amitriptyline (Sarotex, Tryptizol)
- Clomipramine (Anafranil)
- Imipramine en Nortiptyline (Notrilen)
- Paroxetine (Seroxat)
- Fluvoxamine (Fevarin)
- Sertraline (Zoloft)
- Anti-Epileptica – Carbamazepine (Carbymal, Tegretol)
- Nifedipine (Adalat)
- Zinksulfat
- Lidocaine
- Gentamicine
- Ginkgo Biloba (Tavonin)

only if you are taking prescribed medication
31. which medicines do you take? Please tick: (list not translated)

32. Verwenden Sie aufgrund der Tinnitusbeschwerden andere verschreibungspflichtige Medikamente?

- Ja, nämlich ...
- Nein

do you use any other prescription medication due to the tinnitus symptoms?
o Yes, namely ...
o No

33. Haben Sie in den letzten drei Monaten aufgrund der Tinnitusbeschwerden ein Hörgerät verwendet?

- Ja, nämlich ...
- Nein

have you used a hearing aid in the last three months because of the tinnitus symptoms?
o Yes, namely ...
o No

34. Haben Sie in den letzten drei Monaten aufgrund der Tinnitusbeschwerden einen Noiser verwendet?

- Ja, nämlich ...
- Nein

Have you used a noiser in the last three months because of the tinnitus symptoms?
o Yes, namely ...
o No

35. Wie oft haben Sie in den letzten drei Monaten aufgrund der Tinnitusbeschwerden einen Homöopathen aufgesucht?

Anzahl der Besuche: ...

wenn Sie keinen Kontakt gehabt haben, geben Sie bitte 0 ein.

how often have you visited a homeopath in the last three months because of the tinnitus complaints?
Number of visits: ...
if you have had no contact, please enter 0.

nur, wenn Sie Kontakt gehabt haben:

36. Wie sind Sie in der Regel zum Homöopathen gekommen?

- Auto
- Fahrrad oder zu Fuß
- Öffentliche Verkehrsmittel
- andere

only if you have had contact:
how did you usually get to the homeopath?
o Car
o Bicycle or on foot
o Public transport
o Other

37. Wie hoch waren in den letzten drei Monaten die Gesamtkosten der Kontakte mit dem Homöopathen?

Kosten in €: ...

Wenn Sie keinen Kontakt gehabt haben, geben Sie bitte 0 ein.

what was the total cost of contacts with the homeopath in the last three months?
Costs in €: ...
If you have had no contact, please enter 0.

38. Wie oft haben Sie in den letzten drei Monaten aufgrund der Tinnitusbeschwerden einen Akupunkteur aufgesucht?

Anzahl der Besuche: ...

wenn Sie keinen Kontakt gehabt haben, geben Sie bitte 0 ein.

how often have you visited an acupuncturist in the last three months because of the tinnitus complaints?
Number of visits: ...
if you have had no contact, please enter 0.

nur, wenn Sie Kontakt gehabt haben:

39. Wie sind Sie in der Regel zum Akupunkteur gekommen?

- Auto
- Fahrrad oder zu Fuß
- Öffentliche Verkehrsmittel
- andere

only if you have had contact:
how did you usually get to the acupuncturist?
o Car
o Bicycle or on foot
o Public transport
o Other

40. Wie hoch waren in den letzten drei Monaten die Gesamtkosten der Kontakte mit dem Homöopathen?

Kosten in €: ...

Wenn Sie keinen Kontakt gehabt haben, geben Sie bitte 0 ein.

what was the total cost of contacts with the homeopath in the last three months?
Costs in €: ...
If you have had no contact, please enter 0.

41. Wie oft haben Sie in den letzten drei Monaten aufgrund der Tinnitusbeschwerden einen Physiotherapeuten aufgesucht?

Anzahl der Besuche: ...

wenn Sie keinen Kontakt gehabt haben, geben Sie bitte 0 ein.

how often have you visited a physiotherapist in the last three months because of the tinnitus complaints?
Number of visits: ...
if you have had no contact, please enter 0.

nur, wenn Sie Kontakt gehabt haben:

42. Wie sind Sie in der Regel zum Physiotherapeuten gekommen?

- o Auto
- o Fahrrad oder zu Fuß
- o Öffentliche Verkehrsmittel
- o andere

only if you have had contact:
how did you usually get to the physiotherapist?
o Car
o Bicycle or on foot
o Public transport
o Other

43. Wie hoch waren in den letzten drei Monaten die Gesamtkosten der Kontakte mit dem Physiotherapeuten?

Kosten in €: ...

Wenn Sie keinen Kontakt gehabt haben, geben Sie bitte 0 ein.

what was the total cost of contacts with the physiotherapist in the last three months?
Costs in €: ...
If you have had no contact, please enter 0

44. Wie oft haben Sie in den letzten drei Monaten aufgrund der Tinnitusbeschwerden einen Hypnotiseur, Wunderheiler oder Parapsychologen aufgesucht?

Anzahl der Besuche: ...

wenn Sie keinen Kontakt gehabt haben, geben Sie bitte 0 ein.

how often have you visited a hypnotist, faith healer or parapsychologist in the last three months because of the tinnitus complaints?
Number of visits: ...
if you have had no contact, please enter 0

nur, wenn Sie Kontakt gehabt haben:

45. **Wie sind Sie in der Regel zum Hypnotiseur, Wunderheiler oder Parapsychologen gekommen?**
    - o Auto
    - o Fahrrad oder zu Fuß
    - o Öffentliche Verkehrsmittel
    - o andere

only if you have had contact:
How did you usually get to the hypnotist, faith healer or parapsychologist?
o Car
o Bicycle or on foot
o Public transport
o Other

46. **Wie hoch waren in den letzten drei Monaten die Gesamtkosten der Kontakte mit dem Homöopathen?**

    Kosten in €: ...

    Wenn Sie keinen Kontakt gehabt haben, geben Sie bitte 0 ein.

what was the total cost of contacts with the homeopath in the last three months?
Costs in €: ...
If you have had no contact, please enter 0.

47. **Haben Sie in den letzten drei Monaten speziell wegen der Tinnitussymptome Sport (z.B. Yoga oder Laufen) getrieben?**

    - o Ja
    - o Nein

have you done any sport (e.g. yoga or running) in the last three months specifically because of the tinnitus symptoms?
o Yes
o No

nur, wenn Sie Sport getrieben haben:

48. **Wie hoch waren in den letzten drei Monaten die Gesamtkosten für Ihre sportlichen Aktivitäten?**

    Kosten in €: ...

    wenn Sie keinen Sport getrieben haben, geben Sie bitte 0 ein.

only if you have done sport:
what was the total cost of your sporting activities in the last three months?
Costs in €: ...
if you did not do any sport, please enter 0.

49. Haben Sie in den letzten drei Monaten eine Form der Meditation vor allem wegen der Tinnitussymptome praktiziert?

- Ja
- Nein

Have you practised any form of meditation in the last three months mainly because of tinnitus symptoms?
o Yes
o No

nur, wenn Sie Meditation praktiziert haben:

50. Wie hoch waren in den letzten drei Monaten die Gesamtkosten für Meditation?

Kosten in €: ...

wenn Sie keine Meditation praktiziert haben, geben Sie bitte 0 ein.

only if you have practised meditation:
What was the total cost of meditation in the last three months?
Costs in €: ...
if you have not practised any meditation, please enter 0.

51. Haben Sie in den letzten drei Monaten eines der folgenden Mittel vor allem aufgrund der Tinnitussymptome verwendet?

Baldrian
- ja, die Kosten betragen für die letzten drei Monate ________€
- nein

Kamille
- ja, die Kosten betragen für die letzten drei Monate ________€
- nein

Ginkgo Biloa
- ja, die Kosten betragen für die letzten drei Monate ________€
- nein

Ohrkerze
- ja, die Kosten betragen für die letzten drei Monate ________€
- nein

have you used any of the following remedies in the last three months mainly because of tinnitus symptoms?
Valerian
o yes, the cost is ________€ for the last three months
o no
Camomile
o yes, the cost for the last three months is ________€

o no
Ginkgo Biloa
o yes, the costs for the last three months amount to ________€
o no
Ear candle
o yes, the costs for the last three months are ________€
o no

52. Wenn Sie in den letzten drei Monaten eine andere Behandlung oder Pflege wahrgenommen, andere Hilfsmittel erhalten oder andere Aktivitäten speziell bzgl. Ihrer Tinnitussymptome unternommen haben, beschreiben Sie diese bitte unten. Bitte nennen Sie auch die damit ggf. verbundenen Kosten.

____
____
____
____
____
____

If you have received any other treatment, care, aids or activities specifically related to your tinnitus symptoms in the last three months, please describe them below. Please also list any costs associated with these activities.

53. Gehen Sie derzeit einer bezahlten Tätigkeit nach?

- Ja
- Nein, STOP

Are you currently in paid employment?
o Yes
o No, STOP

54. Wieviele Stunden pro Woche?

... Stunden pro Woche

How many hours per week?
... Hours per week

55. Über wieviele Tage sind Ihre Arbeitsstunden aufgeteilt?

... Tage

Over how many days are your working hours divided?
... Days

56. Was sind Sie von Beruf?

___

What is your profession?

57. Wie hoch ist Ihr Nettoeinkommen?

Relevant ist nur Ihr eigenes Gesamtnettoeinkommen, nicht das des Partners.

.... € pro Woche
.... € pro 4 Wochen
.... € pro Monat
.... € pro Jahr

- ich weiß es nicht, will ich nicht nennen.

What is your net income?
Only your own total net income is relevant, not that of your partner.
.... € per week
.... € per 4 weeks
.... € per month
.... € per year
o I don't know, I don't want to name.

58. Haben Sie sich während der letzten drei Monate krank gemeldet?

- Ja
- Nein, gehen Sie zu Frage 67

have you taken sick leave during the last three months?
o Yes
o No, go to question 67

59. Sind Sie derzeit krank gemeldet?

- Ja
- Nein, Datum der letzten Abwesenheit?

  ___

Are you currently on sick leave?
o Yes
o No, date of last absence?

60. Wie viele Tage (insgesamt) Sie haben in den letzten drei Monaten gefehlt?

..... Werktage

how many days (in total) you have been absent in the last three months?
..... Working days

61. Wie oft haben Sie die letzten 3 Monate gefehlt?

..... Mal

ow often have you been absent in the last 3 months?
..... Times

62. Wie viele Tage haben Sie während der kürzesten Abwesenheitszeit in den letzten drei Monaten gefehlt?

... Werktage

how many days have you been absent during the shortest period of absence in the last three months?
... Working days

63. Wurde Ihre Arbeit während der Fehltage oder nach kurzer Zeit von jemandem übernommen?

- o Kollegen / Supervisor übernahmen die Arbeit in normalen Zeiten
- o Kollegen / Supervisor übernahmen die Arbeit zusätzlich
- o Zusätzliche Kräfte übernahmen die Arbeit
- o Sie haben die Arbeit selbst später während der normalen Geschäftszeiten nachgeholt
- o Sie haben die Arbeit später durch Überstunden nachgeholt
- o Arbeit wurde nicht übernommen oder nachgeholt
- o Sie wissen nicht, wer die Arbeit übernommen hat

was your work taken over by someone during the days of absence or after a short time?
o Colleagues / supervisors took over the work during normal times
o Colleagues / supervisors took over the work additionally
o Additional staff took over the work
o You took over the work yourself later during normal business hours
o They made up the work later by working overtime
o Work was not taken over or made up
o You do not know who took over the work

64. Haben Sie schon mehrmals in den letzten drei Monaten gefehlt?

- o Ja
- o Nein, gehen Sie zu Frage 67

Have you been absent several times in the last three months?
o Yes
o No, go to question 67

65. Wie viele Tage haben Sie in den letzten drei Monaten während des längsten Abwesenheitszeitraums gefehlt?

... Werktage

how many days have you been absent during the longest period of absence in the last three months?
... Working days

66. Wurde Ihre Arbeit während der Fehltage oder nach längerer Zeit übernommen?

- o Kollegen / Supervisor übernahmen die Arbeit in normalen Zeiten
- o Kollegen / Supervisor übernahmen die Arbeit zusätzlich
- o Zusätzliche Kräfte übernahmen die Arbeit
- o Sie haben die Arbeit selbst später während der normalen Geschäftszeiten nachgeholt
- o Sie haben die Arbeit später durch Überstunden nachgeholt
- o Arbeit wurde nicht übernommen oder nachgeholt
- o Sie wissen nicht, wer die Arbeit übernommen hat

Was your work taken over during the days of absence or after a longer period of time?
o Colleagues / supervisors took over the work during normal times
o Colleagues / supervisors took over the work additionally
o Additional staff took over the work
o You took over the work yourself later during normal business hours
o They made up the work later by working overtime
o Work was not taken over or made up
o You do not know who took over the work

67. Kreisen Sie bitte auf der Skala unten ein, wie viel Arbeitspensum Sie an Ihrem letzten Arbeitstag (vor Krankmeldung) in Ihrer normalen Arbeitszeit im Vergleich zu einem normalen Arbeitstag geleistet haben.

0 bedeutet, dass Sie nichts tun konnten und 10, dass Sie so viel wie normalerweise geleistet haben

Nichts 0 1 2 3 4 5 6 7 8 9 10 Genauso viel wie normal

Please circle on the scale below how much workload you did in your normal working hours on your last working day (before calling in sick) compared to a normal working day.
0 means you could do nothing and 10 means you did as much as you normally do
Nothing 0 1 2 3 4 5 6 7 8 9 10 as much as normally

68. Kreisen Sie bitte auf der Skala unten ein, wie die Qualität der Arbeit einzuschätzen war, die Sie während Ihres letzten Arbeitstag im Vergleich zu einem normalen Arbeitstag geleistet haben.

0 bedeutet, dass Ihre Arbeit von sehr schlechter Qualität war und 10, dass Sie die gleiche Qualität wie normalerweise erbracht haben.

Nichts 0 1 2 3 4 5 6 7 8 9 10 Genauso viel wie normal

Please circle on the scale below how you rated the quality of the work you did during your last working day compared to a normal working day.
0 means that your work was of very poor quality and 10 means that you did the same quality as normal.
Nothing 0 1 2 3 4 5 6 7 8 9 10 as much as normally

69. Hätten Sie bei der Frage 15 oder 16 die Zahl 10 eingekreist?

- nein, wegen der Tinnitusbeschwerden
- nein, wegen anderer gesundheitlicher Probleme
- nein, wegen der Probleme mit der Arbeit (ohne Ausrüstung, Maschie kaputt, etc.)
- nein, wegen anderem Grund, nämlich ....
- ja

would you have circled the number 10 in question 15 or 16?
o no, because of the tinnitus complaints
o no, because of other health problems
o no, because of the problems with work (no equipment, maschie broken, etc.)
o no, because of other reason, namely ....
o yes

70. Was ist Ihr höchster Bildungsabschluss?

- ohne Abschluss
- Mittlerer Schulabschluss MSA / POS
- Fachabitur
- Abitur
- Berufsausbildung / Lehrberuf
- Meister
- Fachhochschule
- Universität

what is your highest educational qualification?
o Without qualification
o Intermediate school leaving certificate MSA / POS
o Fachabitur
o A-levels
o Vocational training / apprenticeship
o Master craftsman
o Technical college
o University

Tinnituszentrum

II.

Dieser Fragebogen enthält Fragen, die sich mit verschiedenen Aspekten Ihrer Gesundheit befassen.
Bitte denken Sie beim Beantworten an Ihren Gesundheitszustand und Ihre Fähigkeiten, alltägliche Dinge zu tun.
Auswirkungen eines leichten Infekts wie sie z.B. durch eine Erkältung entstehen, können bei der Beantwortung der Fragen vernachlässigt werden, da es sich i.d.R. nur um eine kurzzeitige Einschränkung handelt und Sie in kurzer Zeit wieder vollständig genesen.
Bitte beantworten Sie die Fragen im Hinblick auf die Einschränkungen, die Sie in der Regel empfinden.

Nicht alle Fragen sind gleich passend für alle Menschen, es ist im Rahmen dieser Befragung jedoch wichtig, allen die gleichen Fragen zu stellen; auch sich möglicherweise wiederholende Fragen bitten wir Sie zu beantworten.

Lesen Sie jede Frage sorgfältig und prüfen Sie Ihre Antworten. Kreuzen Sie jeweils die Antwort an, die am besten Ihre übliche Situation beschreibt.

Ihre Antworten werden streng vertraulich behandelt. Es gibt keine richtigen oder falschen Antworten; das Ziel ist, das bestmögliche Bild von Ihren Fähigkeiten und Gefühlen zu erhalten.

This questionnaire contains questions that deal with different aspects of your health.
When answering, please think about your state of health and your ability to do everyday things.
Effects of a mild infection, such as those caused by a cold, can be disregarded when answering the questions, as it is usually only a short-term limitation and you will make a full recovery in a short time.
Please answer the questions with regard to the limitations you usually experience.

Not all questions are equally appropriate for all people, but it is important in the context of this survey to ask everyone the same questions; we also ask you to answer any questions that may be repetitive.

Read each question carefully and check your answers. In each case, tick the answer that best describes your usual situation.

Your answers will be kept strictly confidential. There are no right or wrong answers; the aim is to get the best possible picture of your abilities and feelings.

1. Welche der folgenden Aussagen beschreibt am besten, wie gut Sie in der Regel gedruckte Texte lesen oder sehen (z.B. Zeitungen) können?

    a. Kann gut genug sehen, um ohne Brille oder Kontaktlinsen auszukommen.
    b. Gute Sicht mit Brille oder Kontaktlinsen.
    c. Kann mit Brille oder Kontaktlinsen nicht genug sehen.
    d. Kann überhaupt nicht sehen.

which of the following statements best describes how well you can usually read or see printed texts (e.g. newspapers)?

a. Can see well enough to get by without glasses or contact lenses.
b. Good vision with glasses or contact lenses.
c. Cannot see well enough with glasses or contact lenses.
d. Cannot see at all.

2. Welche der folgenden Aussagen beschreibt am besten, wie gut Sie in der Regel einen Freund auf der anderen Straßenseite erkennen können?

    a. Kann gut genug sehen, um ohne Brille oder Kontaktlinsen auszukommen.
    b. Gute Sicht mit Brille oder Kontaktlinsen.
    c. Kann mit Brille oder Kontaktlinsen nicht genug sehen.
    d. Kann überhaupt nicht sehen.

which of the following statements best describes how well you can usually recognise a friend across the road?

a. Can see well enough to get by without glasses or contact lenses.
b. Good vision with glasses or contact lenses.
c. Cannot see well enough with glasses or contact lenses.
d. Cannot see at all.

3. Welche der folgenden Aussagen beschreibt am besten, wie gut Sie normalerweise hören können, was mit mindestens drei anderen Personen in einem Gespräch gesagt wird?

    a. Kann ohne Hörgerät hören, was gesagt wird.
    b. Kann mit Hörgerät hören, was gesagt wird.
    c. Kann auch mit einem Hörgerät nicht hören, was gesagt wird.
    d. Kann nicht hören, was gesagt wird, trage aber kein Hörgerät.
    e. Kann überhaupt nicht hören.

which of the following best describes how well you can usually hear what is being said with at least three other people in a conversation?

a. Can hear what is being said without a hearing aid.
b. Can hear what is being said with a hearing aid.
c. Cannot hear what is being said even with a hearing aid.
d. Cannot hear what is being said but do not wear a hearing aid.
e. Cannot hear at all.

4. <u>Welche der folgenden Aussagen beschreibt am besten, wie gut Sie normalerweise hören können, was in einem Gespräch mit einer anderen Person in einem ruhigen Raum gesagt wird?</u>

    a. Kann ohne Hörgerät hören, was gesagt wird.
    b. Kann mit Hörgerät hören, was gesagt wird.
    c. Kann auch mit einem Hörgerät nicht hören, was gesagt wird.
    d. Kann nicht hören, was gesagt wird, trage aber kein Hörgerät.
    e. Kann überhaupt nicht hören.

which of the following best describes how well you can usually hear what is being said in a conversation with another person in a quiet room?

a. Can hear what is being said without a hearing aid.
b. Can hear what is being said with a hearing aid.
c. Cannot hear what is being said even with a hearing aid.
d. Cannot hear what is being said but do not wear a hearing aid.
e. Cannot hear at all.

5. <u>Welche der folgenden Aussagen beschreibt am besten, wie gut Sie in der Regel verstanden werden, wenn Sie mit Fremden in der eigenen Sprache sprechen?</u>

    a. Werde vollständig verstanden.
    b. Meine Worte werden zum Teil verstanden.
    c. Meine Worte werden nicht verstanden.
    d. Kann überhaupt nicht sprechen.

which of the following statements best describes how well you are usually understood when you speak to strangers in their own language?

a. Be fully understood.
b. My words are partially understood.
c. My words are not understood.
d. Cannot speak at all.

6. <u>Welche der folgenden Aussagen beschreibt am besten, wie gut meinen Sie in der Regel verstanden zu werden, wenn Sie mit Menschen sprechen?</u>

    a. Werde vollständig verstanden.
    b. Meine Worte werden zum Teil verstanden.
    c. Meine Worte werden nicht verstanden.
    d. Kann überhaupt nicht sprechen.

which of the following statements best describes how well you usually think you are understood when you talk to people?

a. Be fully understood.
b. My words are partially understood.
c. My words are not understood.
d. Cannot speak at all.

7. Welche der folgenden Aussagen beschreibt am besten, wie Sie sich in der Regel fühlen?

    a. Glücklich und am Leben interessiert.
    b. Etwas glücklich.
    c. Etwas unglücklich.
    d. Sehr unglücklich.
    e. So unglücklich, dass das Leben nichts wert scheint.

which of the following statements best describes how you usually feel?
a. Happy and interested in life.
b. Somewhat happy.
c. Somewhat unhappy.
d. Very unhappy.
e. So unhappy that life seems worth nothing.

8. Welche der Aussagen beschreibt am besten, wie viel Schmerz und Beschwerden Sie in der Regel haben?

    a. Keine Schmerzen und Beschwerden.
    b. Leichte Schmerzen oder Beschwerden, die jedoch keine Aktivität behindern.
    c. Moderate Schmerzen oder Beschwerden, die einige Aktivitäten behindern.
    d. Mittelschwere bis schwere Schmerzen oder Beschwerden, die einige Aktivitäten behindern.
    e. Starke Schmerzen oder Beschwerden, die die meisten Aktivitäten behindern.

which of the statements best describes how much pain and discomfort you usually experience?
a. No pain and discomfort.
b. Mild pain or discomfort, but it does not interfere with any activity.
c. Moderate pain or discomfort that interferes with some activities.
d. Moderate to severe pain or discomfort that interferes with some activities.
e. Severe pain or discomfort that interferes with most activities.

9. Welche der folgenden Aussagen beschreibt am besten, wie gut Sie in der Regel gehen können?
   Hinweis: mit Hilfsmitteln meinen wir z.B. Schiene, Stock, Krücke, Prothese, Walker, Rollator etc.

    a. Kann leicht und ohne fremde Hilfe herumlaufen.
    b. Kann mit Mühe in der Nachbarschaft herumlaufen, benötige aber keine Hilfsmittel oder Hilfe von einer anderen Person.
    c. Kann zu Fuß in der Nähe mit einem Hilfsmittel und ohne die Hilfe einer anderen Person herum laufen.
    d. Kann mit einem Hilfsmittel kurzen Wege gehen, benötige aber einen Rollstuhl, um mich weiter zu bewegen

e. Kann nicht selbstständig ohne Hilfsmittel gehen. Mit Hilfe einer anderen Person kann ich kurze Wege gehen, benötige aber einen Rollstuhl, um mich weiter zu bewegen.

which of the following statements best describes how well you can usually walk?
Note: by assistive devices we mean e.g. splint, cane, crutch, prosthesis, walker, rollator etc.

a. Can walk around easily and without assistance.
b. Can walk around the neighbourhood with difficulty, but do not need any aids or help from another person.
c. Can walk around the neighbourhood with an aid and without the help of another person.
d. Can walk short distances with an assistive device, but need a wheelchair to move further.
e. Cannot walk independently without an assistive device. I can walk short distances with the help of another person, but I need a wheelchair to move further.

10. <u>Welche der folgenden Aussagen beschreibt am besten, wie gut Sie in der Regel Ihre Hänge und Finger benutzen können?
Hinweis: damit meinen wir z.B. Knöpfe schließen, Tasten drücken, Werkzeug benutzen, Anheben kleiner Objekte, auch mit Hilfsmitteln, um die Nutzung der Hände und Finger zu unterstützen</u>

a. Volle Nutzung von zwei Händen und zehn Fingern.
b. Einschränkungen bei der Verwendung von Händen oder Fingern, aber Hilfsmittel oder Hilfe einer anderen Person nicht erforderlich.
c. Einschränkungen bei der Verwendung von Händen oder Fingern, kann aber mit Hilfsmitteln selbstständig agieren und benötige keine Unterstützung einer anderen Person.
d. Einschränkung bei der Verwendung der Hände und Finger, benötige die Hilfe einer anderen Person für einige Aufgaben (kann nicht selbstständig agieren, auch nicht mit Hilfsmitteln.
e. Einschränkung bei der Verwendung der Hände und Finger, benötige die Hilfe einer anderen Person für die meisten Aufgaben (kann nicht selbstständig agieren, auch nicht mit Hilfsmitteln.
f. Einschränkung bei der Verwendung der Hände oder Finger, benötige die Hilfe einer anderen Person für alle Aufgaben (kann nicht selbstständig agieren, auch nicht mit Hilfsmitteln.

which of the following statements best describes how well you can usually use your hands and fingers?
Note: by this we mean e.g. closing buttons, pressing keys, using tools, lifting small objects, also using aids to support the use of your hands and fingers

a. Full use of two hands and ten fingers.
b. Restrictions on use of hands or fingers, but aids or help from another person not required.
c. Limitations in the use of hands or fingers, but can act independently with assistive devices and do not require assistance from another person.

d. Limitation in the use of hands and fingers, need the help of another person for some tasks (cannot act independently, even with assistive devices.
e. Restricted in the use of hands and fingers, need the help of another person for most tasks (cannot act independently, even with assistive devices.
f. Restriction in the use of hands or fingers, need the help of another person for all tasks (cannot act independently, even with assistive devices.

11. Welche der folgenden Aussagen beschreibt am besten, wie gut Sie die Dinge normalerweise erinnern können?

    a. Kann mich gut an die meisten Dinge erinnern.
    b. Bin etwas vergesslich.
    c. Bin sehr vergesslich.
    d. Kann mir nichts merken.

Which of the following statements best describes how well you can usually remember things?
a. Can remember most things well.
b. Am somewhat forgetful.
c. Am very forgetful.
d. Can't remember anything.

12. Welcher der folgenden Aussagen beschreibt am besten, wie gut Sie in der Regel denken und alltägliche Probleme lösen können?

    a. Kann klar denken und alltägliche Probleme lösen.
    b. Habe ein wenig Schwierigkeiten zu denken und alltägliche Probleme zu lösen.
    c. Habe einige Schwierigkeiten zu denken und alltägliche Probleme zu lösen
    d. Habe große Schwierigkeiten mit dem Denken und Lösen alltäglicher Probleme.
    e. Kann nicht denken oder alltägliche Probleme lösen.

12. which of the following statements best describes how well you can usually think and solve everyday problems?
a. Can think clearly and solve everyday problems.
b. Have a little difficulty thinking and solving everyday problems.
c. Have some difficulty thinking and solving everyday problems.
d. Have great difficulty thinking and solving everyday problems.
e. Cannot think or solve everyday problems.

13. Welche der folgenden Aussagen beschreibt am besten, wie gut Sie in der Regel tägliche Aktivitäten durchführen können?

    a. Kann normal essen, mich waschen, anziehen und die Toilette benutzen.
    b. Kann selbstständig, aber mit Schwierigkeiten essen, mich waschen, anziehen und die Toilette benutzen.
    c. Benutze Hilfsmittel, um selbstständig zu essen, mich zu waschen, anzuziehen und die Toilette zu benutzen.

d. Benötige Hilfe von einer anderen Person, um zu essen, mich zu waschen, anzuziehen und die Toilette zu benutzen.

Which of the following statements best describes how well you can usually perform daily activities?

a. Can eat, wash, dress and use the toilet normally.
b. Can eat, wash, dress and use the toilet independently but with difficulty.
c. Use aids to eat, wash, dress and use the toilet independently.
d. Need help from another person to eat, wash, dress and use the toilet.

14. <u>Welche der folgenden Aussagen beschreibt am besten, wie Sie sich in der Regel fühlen?</u>

a. Im Allgemeinen fröhlich und sorglos.
b. Gelegentlich traurig, wütend, reizbar, ängstlich oder depressiv.
c. Oft traurig, wütend, reizbar, ängstlich oder depressiv.
d. Fast immer traurig, reizbar, ängstlich oder depressiv.
e. Sehr traurig, wütend, reizbar, ängstlich oder depressiv bis zu dem Punkt, an dem professionelle Hilfe benötigt wird.

which of the following statements best describes how you usually feel?

a. Generally cheerful and carefree.
b. Occasionally sad, angry, irritable, anxious or depressed.
c. Often sad, angry, irritable, anxious or depressed.
d. Almost always sad, irritable, anxious or depressed.
e. Very sad, angry, irritable, anxious or depressed to the point where professional help is needed.

15. <u>Welche der folgenden Aussagen beschreibt am besten, wie viel Schmerzen oder Beschwerden Sie in der Regel haben?</u>

a. Habe keine Schmerzen und Beschwerden.
b. Habe gelegentlich Schmerzen und Beschwerden. Unangenehm ist, wenn sie nicht durch verschriebene Medikamente oder Übungen gelindert werden. Normale Aktivitäten sind nicht beeinträchtigt.
c. Habe oft Schmerzen oder Beschwerden. Unannehmlichkeiten werden durch Einnahme von Medikamenten erleichtert, diese beeinträchtigen jedoch manchmal die normalen Aktivitäten.
d. Habe oft Schmerzen oder Beschwerden. Normale Aktivitäten sind oft beeinträchtigt. Verschreibungspflichtige Medikamente zur Linderung werden benötigt.
e. Habe starke Schmerzen oder Beschwerden, die nicht mit Medikamenten zu lindern sind. Der Schmerz beeinträchtigt ständig die normalen Aktivitäten.

which of the following best describes how much pain or discomfort you usually have?

a. Have no pain or discomfort.
b. Have occasional pain and discomfort. Discomfort is when it is not relieved by prescribed medication or exercise. Normal activities are not affected.

c. Have frequent pain or discomfort. Discomfort is relieved by taking medication, but this sometimes interferes with normal activities.
d. Have frequent pain or discomfort. Normal activities are often affected. Prescription medication for relief is needed.
e. Have severe pain or discomfort that cannot be relieved with medication. Pain constantly interferes with normal activities

16. Wie würden Sie insgesamt Ihren Gesundheitszustand einschätzen?

   a. Ausgezeichnet.
   b. Sehr gut.
   c. Gut.
   d. Mäßig.
   e. Schlecht.

Overall, how would you rate your state of health?
a. Excellent.
b. Very good.
c. Good.
d. Moderate.
e. Poor.

17. Wie haben Sie diesen Fragebogen ausgefüllt? Kreisen Sie die Antwort ein, die Ihre Situation am besten beschreibt.

   a. Selbstständig, ohne Hilfe von einer anderen Person.
   b. Eigenständig; eine andere Person hat geholfen, die Antworten einzutragen.
   c. Mit Hilfe einer anderen Person.
   d. Dieser Fragebogen wurde ohne die Hilfe der betroffenen Person oder des Patienten von einem Familienmitglied ausgefüllt.
   e. Dieser Fragebogen wurde von einer Krankenschwester oder einer pflegenden Person ohne die Mitwirkung der betroffenen Person oder des Patienten ausgefüllt. Bitte geben Sie Ihre Funktion an:
      ____
   f. Dieser Fragebogen wurde von einer anderen Person ohne die Mitwirkung der betroffenen Person oder des Patienten ausgefüllt. Bitte geben Sie Ihre Beziehung zur betroffenen Person oder des Patienten an:
      ____

How did you fill in this questionnaire? Circle the answer that best describes your situation.
a. Independently, without help from another person.
b. Independently; another person helped to fill in the answers.
c. With the help of another person.
d. This questionnaire was filled in by a family member without the help of the person concerned or the patient.
e. This questionnaire was completed by a nurse or carer without the involvement of the person concerned or the patient. Please indicate your function:
   ____

f. This questionnaire was completed by another person without the involvement of the person concerned or the patient. Please indicate your relationship to the person concerned or the patient: ________________________________

Vielen Dank für das Ausfüllen des Fragebogens.

Thank you for completing the questionnaire.
